# Clinicomycological Characterization of Superficial Mycoses from a Tertiary Care Hospital in Nepal

**DOI:** 10.1155/2016/9509705

**Published:** 2016-11-24

**Authors:** Sundar Khadka, Jeevan Bahadur Sherchand, Dinesh Binod Pokharel, Bharat Mani Pokhrel, Shyam Kumar Mishra, Subhash Dhital, Basista Rijal

**Affiliations:** ^1^HIV Reference Unit, National Public Health Laboratory, Kathmandu, Nepal; ^2^Department of Microbiology, Tribhuvan University Teaching Hospital, Kathmandu, Nepal; ^3^Department of Dermatology, Tribhuvan University Teaching Hospital, Kathmandu, Nepal

## Abstract

*Background*. Superficial mycosis is a common fungal infection worldwide, mainly caused by dermatophytes. However, the prevalence of species varies geographically. In addition, fungal treatment is best guided according to species isolated. This study was carried out to determine the clinical as well as mycological profile of superficial mycoses in a tertiary care hospital, Nepal.* Methods*. This was a prospective case-control laboratory based study conducted over a period of six months from January to June 2014 at Tribhuvan University Teaching Hospital, Nepal. A total of 200 specimens were collected from the patients suspected of superficial mycoses. The specimens were macroscopically as well as microscopically examined. The growth was observed up to 4 weeks.* Results.* Out of total 200 specimens from the patients suspected of superficial mycoses, tinea corporis 50 (25%) was most common clinical types. KOH mount was positive in 89 (44.5%) and culture was positive in 111 (55.5%).* Trichophyton mentagrophytes* 44 (39.6%) was the most common isolate.* Conclusions.* The diagnostic yields of KOH mount and culture were found to be complementary to each other. Thus both the methods added with clinical findings are equally important to establish superficial mycosis.

## 1. Introduction 

Superficial mycosis is a disease of the skin and its appendages caused by fungi. It comprises dermatophytosis, candidiasis, and pityriasis versicolor [1]. They have the affinity to keratin rich tissues and produce dermal inflammatory response, intense itching, and cosmetically poor appearance [1]. Superficial mycoses are common worldwide [2]. They are thought to affect 20% to 25% of the world's population, and the incidence of superficial mycoses continues to increase [2]. An etiological agent of superficial fungal infections consists of dermatophytes, yeasts, and nondermatophyte molds [3]. Dermatophyte is responsible for most superficial fungal infection and the expected lifetime risk of getting a dermatophyte infection is between 10 and 20% [4].

The dermatophytes are a group of closely related fungi infecting skin, hair, and nails in living host including man. They produce an infection called dermatophytosis, also known as ringworm or tinea [5, 6]. The skin infections caused by nondermatophytic fungi are known as dermatomycoses whereas hair and nail are known as piedra and onychomycosis, respectively [2].

Clinically, tinea can be classified depending on the site of involvement including tinea capitis, tinea corporis, tinea cruris, tinea pedis, and tinea barbae [2]. Invasion of the nail plate by a dermatophyte is referred to as tinea unguium; infection of the nail by nondermatophytic fungi is called onychomycosis. Nowadays, onychomycosis represents the general term to any fungal nail infection [7].

Candidiasis is caused by* Candida* spp. and comprises infections that range from superficial skin infections and mucosal membranes to systemic and potentially life-threatening condition [8]. Pityriasis versicolor (tinea versicolor) is a common, recurrent, superficial fungal infection of stratum corneum which is caused by* Malassezia furfur* [9].

This study was carried out to characterize the clinical pattern and their etiology from suspected cases. Though there are many studies available from across India and world, there are very little data of superficial mycoses from Nepal. Identification of dermatophytes at species level in clinical diagnosis is important not only for epidemiological study but also for antifungal treatment [10].

## 2. Materials and Methods

This was a prospective case-control laboratory based study conducted over a period of six months from January to June 2014. A total of 200 clinical specimens from the patients suspected of superficial mycoses were collected at Department of Dermatology and specimens were processed at Department of Clinical Microbiology of Tribhuvan University Teaching Hospital (TUTH) for direct microscopic examination (KOH mount) and fungal culture. Samples were collected after disinfecting skin surface with 70% alcohol. The samples were collected from the edge of lesion. Hair plucking was collected after selecting infected area, removing at least 10 hairs and scraped scalp scales if present. Nail samples were collected by scrapping infected nail area, or clip infected nail. Samples were transported between two clean glass slides taped together as per standard protocol [10].

All specimens were analyzed for KOH mount and inoculated onto three sets of culture media including Sabouraud Dextrose Agar (SDA) containing chloramphenicol (0.05%) with and without cycloheximide (0.5%) and Dermatophyte Test Medium (DTM) to grow dermatophyte. Cycloheximide in agars was used to isolate the dermatophyte by inhibiting several fungi, including* Aspergillus* and the mucoraceous moulds* Rhizopus*,* Absidia*, and* Mucor* [10]. Culture media were incubated at 25 and 37°C up to 4 weeks. After incubation, these tubes were observed daily for one week and thereafter twice weekly. When growth of fungi was observed, subculture was done by stab inoculation on potato dextrose agar (PDA) plate to stimulate sporulation and incubated at the appropriate temperature until there were sufficient growths for identification.

Repeat cultures were performed in cases where culture was negative for dermatophytes but positive for nondermatophyte moulds or yeasts to rule out the possibility of contamination. Confirmed diagnosis of NDM (nondermatophyte moulds) was performed based on following criteria: (i) abnormality consistent with superficial mycoses, (ii) positive KOH preparation, the presence of filamentous fungi in biological fluid material, (iii) failure to isolate a dermatophyte culture, and (iv) the growth of nondermatophyte moulds in three successive occasions at least, with a minimum of 2-week interval [11].

All cultures were evaluated both macroscopically and microscopically under lactophenol cotton blue (LPCB) mount using cellophane tape preparation, tease mount preparation, and slide culture techniques to detect the formation of macroconidia and microconidia or other typical morphological forms of fungi (Figures 2, 3, 4, 5, and 6). Christensen's urea agars, hair perforation tests, and pigment production on PDA were used to differentiate between* T. mentagrophytes *and* T. rubrum*. The morphology of the fungi was compared to color atlas of reference text books and identified [12, 13]. The cultured agars were incubated for 4 weeks before declaring the culture result as negative [10].

## 3. Results

**Figure 1:**
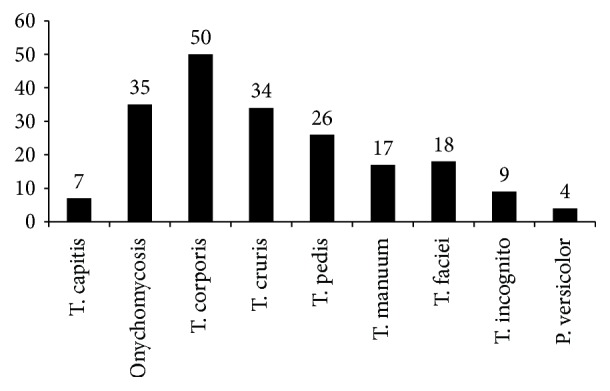
Distribution of various clinical types (*N* = 200).

**Figure 2:**
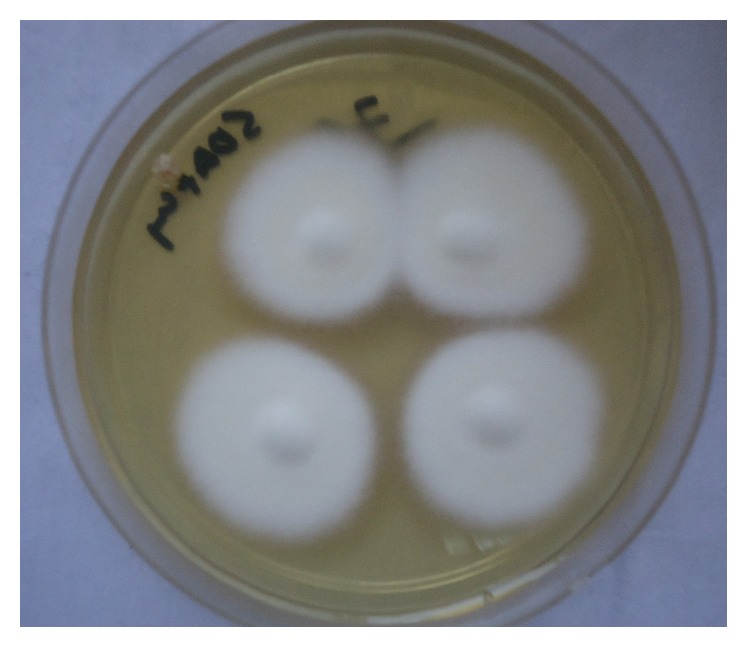
Macroscopic view of* T. mentagrophytes* (forward view).

**Figure 3:**
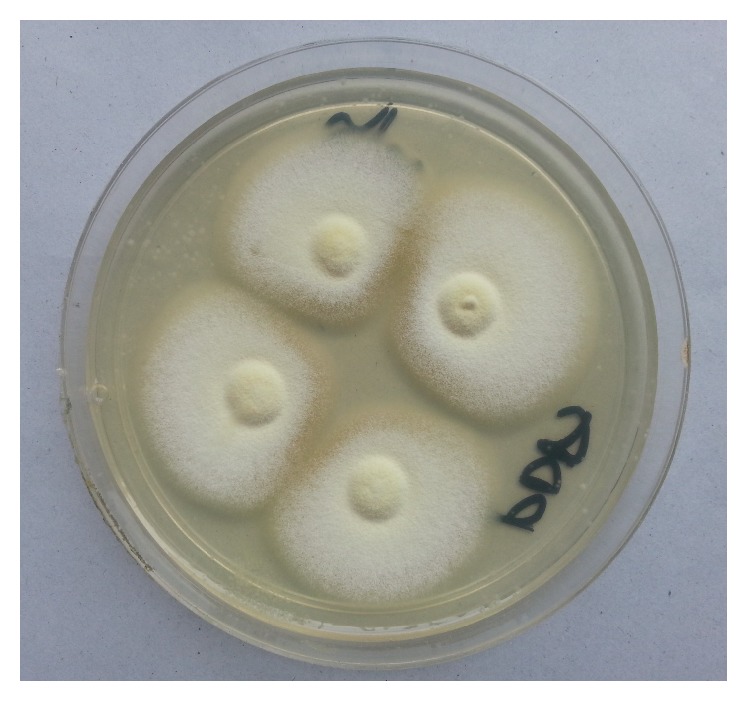
Macroscopic view of* M. canis* (forward view).

**Figure 4:**
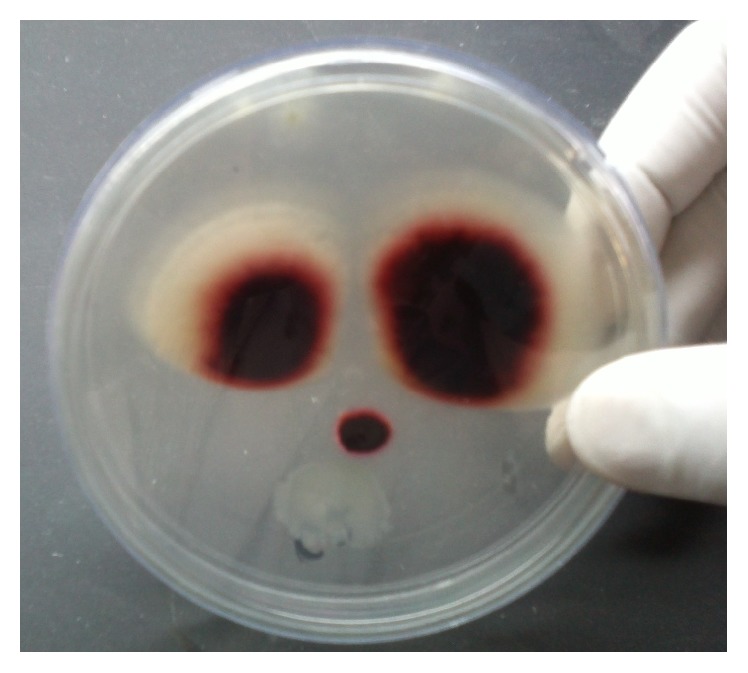
Macroscopic View of* T. rubrum* (reverse view).

**Figure 5:**
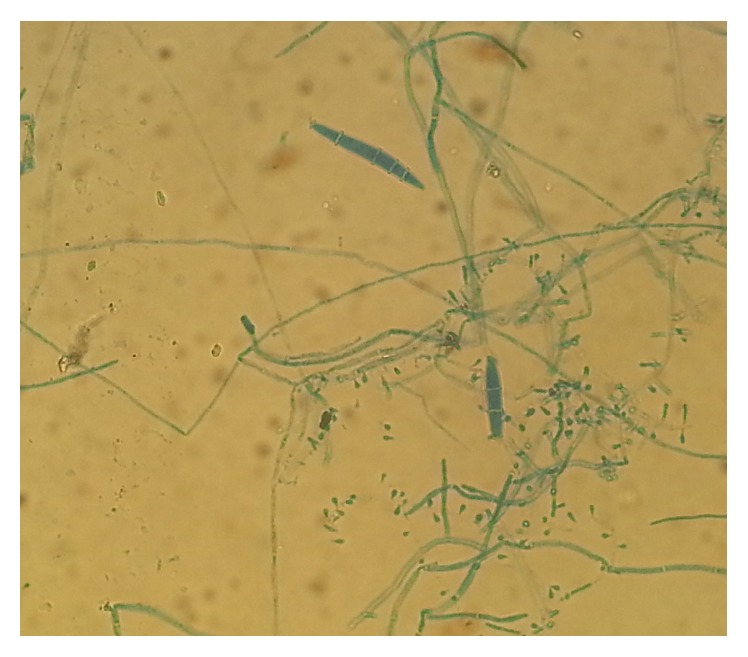
Microscopic view of* M. canis* (LPCB mount, 400x).

**Figure 6:**
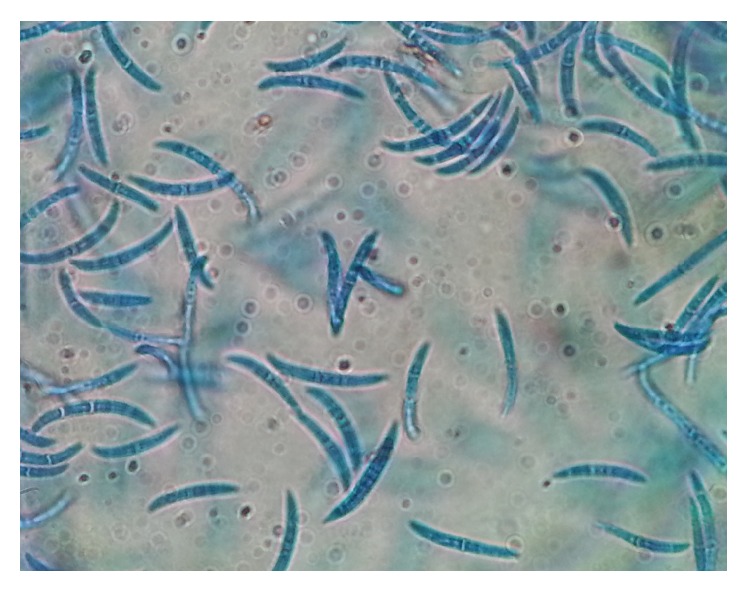
Microscopic view of* Fusarium *spp. (LPCB mount, 400x).

Out of 200 specimens studied, t. corporis (50) (25%) was the most common clinical type followed by onychomycosis (35) (17.5%), t. cruris (34) (17%), and t. pedis (26) (13%), respectively, as shown in Figure 1. 159 skin scrapping, 34 nail clipping, and 7 hair plucking samples from total 200 specimens were examined. Direct microscopy KOH mount was positive in 89 (44.5%) and culture was positive in 111 (55.5%) cases. KOH positive was seen in 55.9% of nail clipping and 44% of skin scrapping while in hair plucking there was no KOH positivity. Growth was seen in 56% of skin scrapping, 52.9% of nail clipping, and 57.1% of hair plucking.

KOH positive with culture positive was seen in 63 (31.5%) cases. KOH positive with culture negative was seen in 26 (13.0%). KOH negative with culture positive was seen in 48 (24.0%). KOH negative with culture negative was seen in 63 (31.5%) as shown in Table 1. Among the 111 culture positive isolates 72 (64.9%) dermatophytes, 31 (27.9%) nondermatophytes (NDM), and 8 (7.2%) yeasts were isolated. Among the dermatophytes, 44 (39.6)* T. mentagrophyte*, 13 (11.7%)* T. rubrum*, 6 (5.4%)* T. tonsurans*, 6 (5.4%)* M. canis*, and 3 (2.7%)* E. floccosum *were isolated. Among the nondermatophyte fungi, 16 (14.4%)* Aspergillus *spp., 5 (4.5%)* Cladosporium*, 4 (3.6%)* Scopulariopsis*, 2 (1.8%)* Fonsecaea*, 2 (1.8%)* Penicillium* spp., 1 (0.9%)* Bipolaris*, and 1 (0.9%)* Fusarium* were isolated. In another group of fungi 8 (7.2%)* Candida* spp. were isolated as shown in Table 2.

*Trichophyton mentagrophytes* (44) (39.6) was the most common fungal pathogen isolated from all clinical types of superficial mycoses. The details about clinicomycological characterization of superficial mycoses were given in Table 3. Superficial mycoses were more common in males (155) (77.5%) and less common in females (45) (22.5%). Male to female ratio was 3.4 : 1. Majority growth of fungi (39) (35.14%) was isolated from age group 21–30 followed by 20 (18.2%) from age group 11–20 and 18 (16.22%) from age group 31–40, respectively, as shown in Table 4.

## 4. Discussion

An accurate diagnosis of superficial mycoses is based on KOH mount and fungal culture. Identification of fungus at species level is helpful for treatment purpose. In this study, KOH positive rate was 44.5% and culture positive rate was 55.5% which was similar to findings of Grover et al. (53%, 79.1%) and Sen et al. (49%, 51%) [1, 14]. The KOH positivity rate varied from 35.6% to 88.6% in various studies and the culture positivity rate from 36% to 53.6%. In these studies, the proportion of KOH negative isolates turning positive on culture varied widely from 5.6% to 56.7% [15].

In this study, 48 (24%) cases were negative for fungal elements in KOH mount but culture positive. So, detection rate of fungal culture (55.5%) was higher than direct microscopy using KOH preparation (44.5%). This may due to the drying procedure advocated by Milne and similar result was found in current study [16]. Our study also correlated with results of Madhavi et al. in 2011 that showed 43% KOH positive and 58% culture positive [17]. These results highlight the importance of culture as well as KOH mount for accurate diagnosis of superficial mycoses. However, Aggarwal et al., Patel et al., and Nawal et al. had reported KOH positive rate (59.20%, 62.12%, and 72.40%, resp.) was greater than culture positive rate (20.15%, 29.29%, and 62.80%), respectively [18–20]. The failure of growth of fungus in a significant proportion of cases was probably due to use of antifungal agent before specimen collection and lack of standard methods for identification of fungus [21].

Our finding showed that tinea corporis was the most common clinical type. Venkatesan et al. also reported in their study that t. corporis was the most common clinical type (64.8%, 24.5%, and 60%, resp.) [22–24]. Other studies done by Suman MN et al. and Sumana V et al. from India also reported that t. corporis was the most common clinical type (48.66% and 60%, resp.) [25, 26].

Out of 200 specimens, 111 specimens show the fungal growth on the fungal culture. Among 111 isolates, majority growths of 72 (64.9%) isolates were dermatophyte, 31 (27.9%) isolates were nondermatophytes moulds (NDM), and 8 (7.2%) isolates were yeasts. Similarly, Prasad et al. in 2013 reported that majority growth of 105 (92.10%) isolates was dermatophyte among the 114/164 cases, of 5(4.38%) isolates was nondermatophytes, and of 4 (3.50%) isolates was yeasts [27].

*Trichophyton* species have been isolated with increasing incidence as compared to* Microsporum* and* Epidermophyton* species [28]. In Asia,* T. rubrum *and* T. mentagrophytes* were most commonly isolated dermatophytes from superficial mycoses [20]. In this study, out of 200 specimens of suspected superficial mycoses,* Trichophyton* (63) (56.8%) was the most frequently isolated genus, with* T. mentagrophytes* (44) (39.7%) as most common species followed by the* T. rubrum* (13) (11.8%).* T. rubrum* were differentiated from* T. mentagrophytes* based on urea hydrolysis test,* in vitro* hair perforation test, pigment production on potato dextrose agar, and macroscopic observation. The present study also correlated with results of Pakshir et al. that showed* T. mentagrophyte* was most common isolate (32.5%, 25%, and 36.8%, resp.) [29–31]. However, Aggarwal A et al., Patel P et al., and Nawal P et al. had reported* T. rubrum* as the most common isolate [18–20]. This may be due to variation in environmental condition and geographical distribution [1].

Our finding showed that, among the nondermatophytes 31/111 (27.9%) cases,* Aspergillus* spp. (16) (14.5%) were the most common isolates followed by* Cladosporium* spp. (5) (4.5%) and* Scopulariopsis* (4) (3.6%). Prasad et al. in 2013 showed that* Aspergillus* spp. (35.1%) were the most common isolates among the nondermatophytes [26] NDM infections on skin, though pathogenic role is not certain yet. The possibility of secondary infection has been raised in various research studies [1, 17]. In our study, we have isolated NDM on skin with certainty using standard mycological technique. In similar ways, various studies as conducted by Grover et al. also reported NDM from skin infections. In these grounds, though the matter of further elucidation, the NDM isolation on skin can be considered [1, 32, 33].

Superficial mycoses were more common in males (77.5%) than in females (22.5%). Male to female ratio was 3.4 : 1. Similar study, conducted by Grover and Roy in 2003, reported superficial mycoses more in males (81%) and less in females (19%) and male to female ratio was 4.2 : 1 [1]. Other studies conducted by Hitendra et al. also supported our study that incidences of superficial mycoses were more prevalent in male (68.16% and 67.5%, resp.) [34, 35]. According to Philpot, males may be more vulnerable to infection than females probably due to higher exposure to infection in the schools and in public bath and sporting activities and use of closed type footwear [36].

In this study, superficial mycosis was more common in the age group 21–30 years (37%) which is comparable with other studies conducted by Lyngdoh et al. (34.4%), Sumana and Rajagopal (52%), and Sen and Rasul (44%) [14, 25, 37]. This may be due to increased physical activity, increased opportunity for exposure, and changes in hormonal pattern [38].

## 5. Conclusion

Clinical finding, KOH mount, and culture reports were found to be complementary to each other in the diagnosis of superficial mycoses. Any clinical diagnosis needs to be supported by laboratory diagnosis. Culture is a necessary adjunct to direct microscopic examination for definitive identification of etiological agent. The treatment of fungal infection would be more effective when antifungal therapy is based on identification of fungal isolates.

## Figures and Tables

**Table 1:** Comparison of KOH mount with fungal culture (*N* = 200).

	Culture, growth	Culture, no growth	Total
KOH positive	63 (31.5%)	26 (13.0%)	89 (44.5%)
KOH negative	48 (24.0%)	63 (31.5%)	111 (55.5%)
Total	111 (55.5%)	89 (44.5%)	200 (100%)

**Table 2:** Frequency of fungal isolates growth (*N* = 111).

Fungi	Number of fungal isolates (*N*)	Number of fungal isolates (%)
Dermatophytes		
*T. mentagrophyte*	44	39.6
*T. rubrum*	13	11.7
*T. tonsurans*	6	5.4
*M. canis*	6	5.4
*E. floccosum*	3	2.7
Nondermatophyte fungi		
*Aspergillus* spp.	16	14.4
*Cladosporium*	5	4.5
*Scopulariopsis*	4	3.6
*Fonsecaea*	2	1.8
*Penicillium *spp.	2	1.8
*Bipolaris*	1	0.9
*Fusarium*	1	0.9
Yeast		
*Candida* spp.	8	7.2
Total	111	100.0

**Table 3:** Clinicomycological characterization of superficial mycoses.

Fungi	Clinical types of superficial mycoses								Total
	*T. capitis *(*N* = 7)	Onychomycosis (*N* = 35)	*T. corporis *(*N* = 50)	*T. cruris *(*N* = 34)	*T. pedis *(*N* = 26)	*T. manuum *(*N* = 17)	*T. faciei *(*N* = 18)	*T. incognito *(*N* = 9)	
*T. mentagrophyte*	1	2	16	9	5	3	6	2	44 (39.6%)
*T. rubrum*	0	3	1	5	2	2	0	0	13 (11.7%)
*T. tonsurans*	0	0	2	1	0	2	1	0	6 (5.4%)
*M. canis*	0	0	0	3	0	0	2	1	6 (5.4%)
*E. floccosum*	0	1	0		0	1	0	0	3 (2.7%)
*Aspergillus *spp.	2	6	6	0	0	1	1	0	16 (14.4%)
*Candida *spp.	1	2	2	1	1	1	0	0	8 (7.2%)
*Scopulariopsis*	0	1	0	0	2	1	0	0	4 (3.6%)
*Cladosporium*	0	2	2	0	1	0	0	0	5 (4.5%)
*Penicillium *spp.	0	1	1	0	0	0	0	0	2 (1.8%)
*Bipolaris*	0	0	0	0	1	0	0	0	1 (0.9%)
*Fusarium*	0	1	0	0	0	0	0	0	1 (0.9%)
*Fonsecaea*	0	0	1	0	0	1	0	0	2 (1.8%)
Total	4 (3.6%)	19 (17.1%)	31 (27.9%)	20 (18.0%)	12 (10.8%)	12 (10.8%)	10 (9.0%)	3 (2.7%)	111 (100%)

**Table 4:** Pattern of fungal growth according to age groups.

Age (years)	Culture		Total
	Growth	No growth	
0–10	7	3	10
11–20	20	14	34
21–30	39	35	74
31–40	18	17	35
41–50	12	8	20
51–60	11	5	16
61–70	4	7	11
Total	111	89	200

## List of Abbreviations

KOH mount: Potassium hydroxide mount
SDA: Sabouraud Dextrose Agar
DTM: Dermatophyte Test Medium
PDA: Potato dextrose agar
LPCB: Lactophenol cotton blue
T. corporis: Tinea corporis
NDM: Nondermatophyte
*T. mentagrophyte*: * Trichophyton mentagrophyte*
*C. albicans*: * Candida albicans*.

## Data Access

The raw data of excel sheet and picture of fungi will be available on request.
